# Functional outcome and quality of life following resection of the proximal humerus performed for musculoskeletal tumors and reconstruction done by four different methods

**DOI:** 10.1007/s12306-022-00771-w

**Published:** 2023-01-17

**Authors:** I. Antal, G. Szőke, M. Szendrői, K. Szalay, T. Perlaky, J. Kiss, G. Skaliczki

**Affiliations:** Department of Orthopedics, Semmelweis University, Üllői út 26, Budapest, 1085 Hungary

**Keywords:** Tumor, Proximal humerus, Reconstruction, Autologous fibula, Bone allograft, Endoprosthesis, Reverse shoulder prosthesis-allograft composite

## Abstract

**Introduction:**

The proximal humerus is a frequent site for both primary and secondary bone tumors. Several options are currently available to reconstruct the resected humerus, but there is no consensus regarding optimal reconstruction. The aim of this retrospective study was to compare the functional outcome, complications and patient compliance following four different types of reconstructive techniques.

**Material and methods:**

The authors performed 90 proximal humerus resections due to primary and secondary bone tumors over the past 21 years. Four different procedures were performed for reconstruction following the resection: fibula autograft transplantation, osteoarticular allograft implantation, modular tumor endoprosthesis (hemiarthroplasty) and reconstruction of the defect with a reverse shoulder prosthesis-allograft composite. A retrospective analysis of the complications and patient’s physical status was performed. Functional outcome and life quality was evaluated by using the MSTS and SF-36 scores.

**Results:**

The best range of motion was observed following arthroplasty with a reverse shoulder prosthesis-homograft composite followed by a fibula autograft reconstruction. Revision surgery was required due to major complications most frequently in the osteoarticular allograft group, followed by the reverse shoulder prosthesis-allograft composite group, the autologous fibula transplantation group; the tumor endoprosthesis hemiarthroplasty group had superior results regarding revision surgery (40, 25, 24 and 14% respectively). MSTS was 84% on average for the reverse shoulder prosthesis-allograft composite group, 70% for the autologous fibula group, 67% for the anatomical hemiarthroplasty group and 64% for the osteoartricular allograft group. Using the SF-36 questionnaire for assessment no significant differences were found between the four groups regarding quality of life.

**Discussion:**

Based on the results of our study the best functional performance (range of motion and patient compliance) was achieved in the a reverse prosthesis-allograft combination group—in cases where the axillary nerve could be spared. The use of an osteoarticular allograft resulted in unsatisfying functional results and high complication rates, therefore we do not recommend it as a reconstructive method following resection of the proximal humerus due to either primary or metastatic bone tumors. Young patients who have good life expectancy but a small humerus or intramedullar cavity reconstruction by implantation of a fibula autograft is a good option. For patients with a poor prognosis (i.g. bone metastases) or in cases where the axillary nerve must be sacrificed, hemiarthroplasty using a tumor endoprosthesis was found to have acceptable results with a low complication rate. According to the MSTS and SF-36 functional scoring systems patients compliance was nearly identical following all four types of reconstruction techniques; the underlying cause may be the complexity of the shoulder girdle. However, we recommend the implantation of a reverse shoulder prosthesis-allograft whenever indication is appropriate, as it has been demonstrated to provide excellent functional outcomes, especially in young adults.

## Introduction

The proximal humerus is frequently targeted by both primary and secondary malignant bone tumors [1, 2]. Limb salvage procedures often compromise both stability and function of the shoulder by excising bone, joint capsule and the tendons of the rotator cuff [1–7]. Several reconstructive options have been proposed following tumor resection [5, 8–14]. The aim of our retrospective study was to compare the functional outcome, complications and patients compliance following four different reconstructive procedures, i.e. hemiarthroplasty with modular tumor endoprosthesis, implantation of a reverse shoulder prosthesis-allograft composite, reconstruction with autologous fibula, and reconstruction with a massive osteoarticular allograft. We hypothesized that reverse shoulder arthroplasty-allograft composite reconstruction would provide better function even in the absence of the rotator cuff and would result in higher patient satisfaction and compliance. Our results were compared to those found in the corresponding literature [3, 10, 14–21].

## Patients and methods

Ninety patients operated between 1999 and 2020 were included in the study, their data were recorded in the files of the Bone Tumor Register of the Department of Orthopedics Semmelweis University. Sixty-one patients were diagnosed with a primary bone tumor, and 29 had surgery for a bone metastasis. Inclusion criteria was patients with a primary or metastatic bone tumor in the proximal third of the humerus without involvement of the glenohumeral joint. Patients who did not meet the criteria for limb-salvage (the tumor involved at least two the major nerves, the entire deltoid muscle and the overlapping skin) or did not survive the first post-operative year, and those who were lost to follow-up for any other reason were excluded from the study.

Average age of the primary bone tumor group was 32 years (10–73), and 65 years (30–76) in the metastasis group. Average follow-up was 96 months (from 10 to 254 months).

**Fig. 1:**
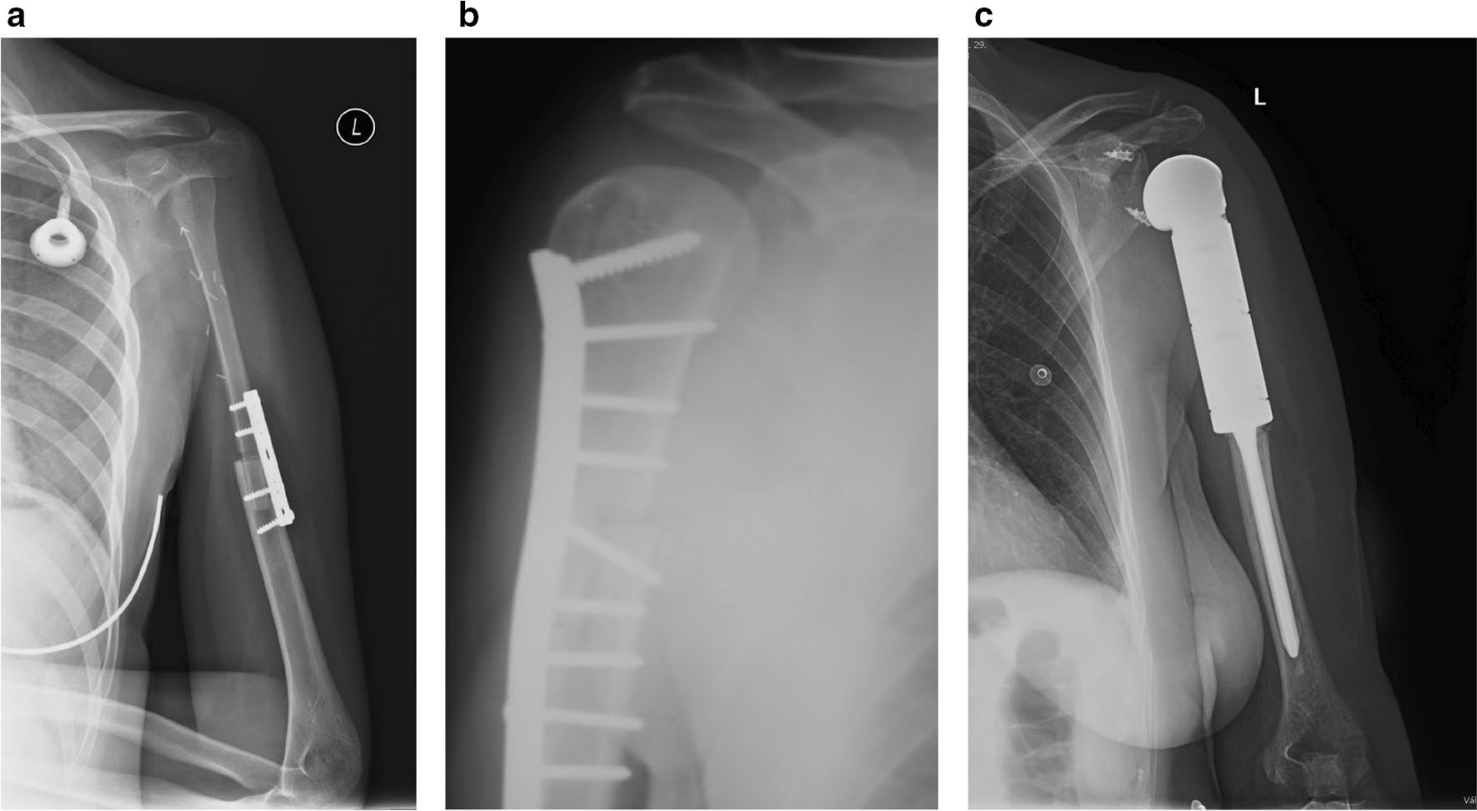
**a** Humerus reconstruction using an autologous fibula. **b** Reconstruction using a massive osteochondral allograft. **c** Hemiarthroplasty with a modular tumor endoprosthesis

**Fig. 2:**
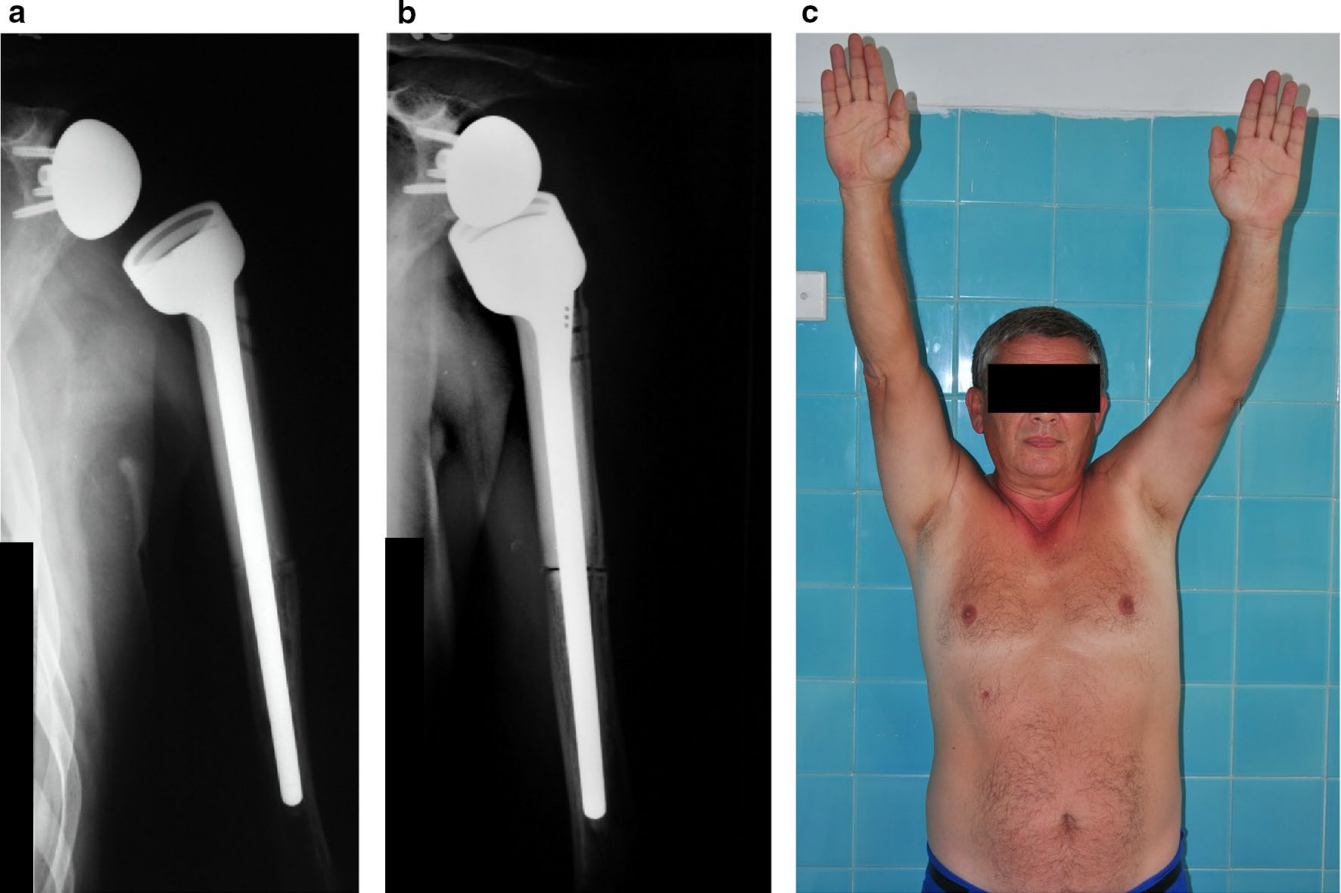
**a** Reconstruction of the left proximal humerus due to a chondrosarcoma using a reverse endoprosthesis-bone allograft. Patient developed instability in the shoulder joint in the post-operative period, so 4 months later the humeral component was augmented with a spacer. **b** Post-operative radiograph at 1 year following the primary procedure, and 8 months following spacer implantation. Deltoid muscle strength increased during the year, so consequentially instability decreased. **c** Good shoulder function and stable joint 1 year following surgery

Primary bone tumors included: osteosarcoma, Ewing’s sarcoma, chondrosarcoma. Malawer I/A type [22] intraarticular resection of the proximal humerus was performed in all 90 cases. Four different surgical procedures were performed for reconstruction:

1. Autologous fibula was transposed to replace the proximal humerus in 25 cases (all but one were non-vascularized grafts) (Fig. 1a),
2. Massive osteoarticular allograft and plate osteosynthesis was performed in 10 cases (Fig. 1b),
3. Anatomical tumor endoprosthesis (hemiarthroplasty) was implanted in 43 cases (Fig. 1c),
4. Reverse shoulder prosthesis-allograft composite reconstruction was done in 12 cases (Fig. 2a).

Indication reading the type of reconstruction following resection was based on the following criteria: biologic reconstruction by autologous fibula was performed in young patients who had good life expectancy and a small humerus with a narrow intramedullar cavity. In middle-age patients with good life expectancy, where the axillary nerve could not be preserved, massive osteochondral homograft was chosen, and reverse shoulder prosthesis-allograft composite was implanted in those with good life expectancy and a preserved axillar nerve. Anatomical tumor endoprostheses were used in patients with short life expectancy or if the rotator cuff and the axillary nerve were sacrificed during resection.

Patient data was reviewed regarding all relevant clinical and radiological documentation. Physical status and MSTS scores were recorded for functional assessment, this included criteria for pain, emotional acceptance, function, ability to position the hand, manual dexterity and ability to lift. Life quality was evaluated using the Short Form-36 (Health Status) [23, 24].

Postoperative complications (i.e. infection, fracture, pseudoarthrosis, joint dislocation, etc.,) were registered and evaluated by the Henderson classification of complications recommended by ISOLS [25].

## Results

The incidence of complications encountered in the four different groups are summarized in Table 1. 25 patients were in the autologous fibula transplantation group and 6 developed complications (24%). All 6 complications were fracture of the graft (Henderson Type 3/B). These responded well to conservative treatment (fixation with an orthosis) in all but one case, where resorption of the graft was observed. A reverse shoulder prosthesis-allograft composite implantation was performed for this patient as a secondary procedure.

**Table 1:** Complication rates according to type of procedure

Complications	Autologous fibular transplantation	Osteoarticular allograft	Tumor endoprosthesis	Reverse prosthesis-allograft composite
Infection	0	1	2	0
Fracture/non-union	6	3	0	0
Dislocation/proximal migration	0	0	4	3
Overall percentage (%)	24	40	14	25

Four complications (40%) occurred in the group of 10 patients who received a massive osteoarticular allograft. Three of these were fractures and collapse of the graft (Henderson Type 3/B) and in one patient developed a late onset post-operative infection (Henderson Type 4/B). In case of one patient who suffered a graft fracture we converted the allograft to a reverse shoulder prosthesis-allograft composite. The infection was successfully managed by graft excision and debridement.

The largest group, including 43 patients, was the tumor endoprosthesis (conventional humerus hemiarthroplasty) group. The overall complication rate was the lowest among all groups, namely 14% (6 cases). Major complications included cranial migration of the prosthesis (1 case), or caudal subluxation with significant instability/dislocation of the prosthesis (5 cases) (Henderson Type 1/A). 1 patient developed a periprosthetic infection (Henderson Type 4/A).

In the reverse shoulder prosthesis-allograft group of 12 patients the only type of complication observed was instability (Henderson Type 1/A). Six cases of luxation occurred within the first 4 postoperative weeks. Closed reduction followed by a 6 week immobilization period and consecutive strengthening of the deltoid muscle led to stability without the need for surgery in 3 cases. Successful revision surgery was performed by lengthening of the humeral component in 2 cases (18%). (Fig. 2a–c).

Only 1 patient had permanent instability despite of revision for over 6 months but he refused surgical treatment and accepted the permanent luxation. The other 5 patients with postoperative luxation had no more shoulder instability after 6 months at the time of follow-up. Infections did not occur in this group.

The four different types of reconstruction following proximal humeral resection lead to different values regarding range of motion in the shoulder joints. Results varied highly in the autologous fibula transplantation group reaching the following range in different directions: flexion 40°–90°, abduction 50°–80°, extension 20°–30°, external rotation 10°–30°, internal rotation 10°–30°. Somewhat lesser values were measured regarding glenohumeral motion in patients who received osteoarticular massive allografts. Limited motion was observed following implantation of tumour prostheses hemiarthroplasty: flexion 40°–60°, abduction: 30°–60°, extension 10°–20°, external rotation10°–20°, internal rotation 10°–30°. In this group, the shoulder joint was stable in 38/43 cases (88%) but there was a 10°–30° lag in joint motion in all directions compared to the fibula transplantation group. These results were unaffected by the use of a Trevira (Implantcast Gmbh) attachment tube.

Shoulder function was found to be worse (no active range of motion) for the 1 patient where the proximal fibula was resorbed.

Best range of motion results were found in the reverse prosthesis-allograft composite group. Average flexion was 120°–170°, abduction−elevation 140°–160°, and extension 20°–30°, internal and external rotation was 10°–30°.

MSTS score was 70% in the autologous fibula transplantation group. The patients in the tumor endoprosthesis group and the massive osteoarticular allograft group achieved similar, though somewhat lower scores: 67 and 64% respectively. The best scores were found in the reverse shoulder prosthesis—bone allograft composite group: 12 patients, 84%. (Table 2).

**Table 2:** Functional outcome assessment based on the MSTS scoring system

MSTS scoring categories	Autologous fibular transplantation	Osteoarticular allograft	Tumor endoprosthesis	Reverse prosthesis-allograft composite
Pain	3.9	4.3	4.7	4.4
Emotional acceptance	3.6	3.4	3.7	4
Function	3.5	3.2	3.1	4.1
Hand positioning	2.9	1.9	1.7	4.2
Manual dexterity	3.9	4	4.6	4.5
Lifting ability	3.5	2.3	2.2	4.1
Total score (%)	70	64	67	84

**Fig. 3:**
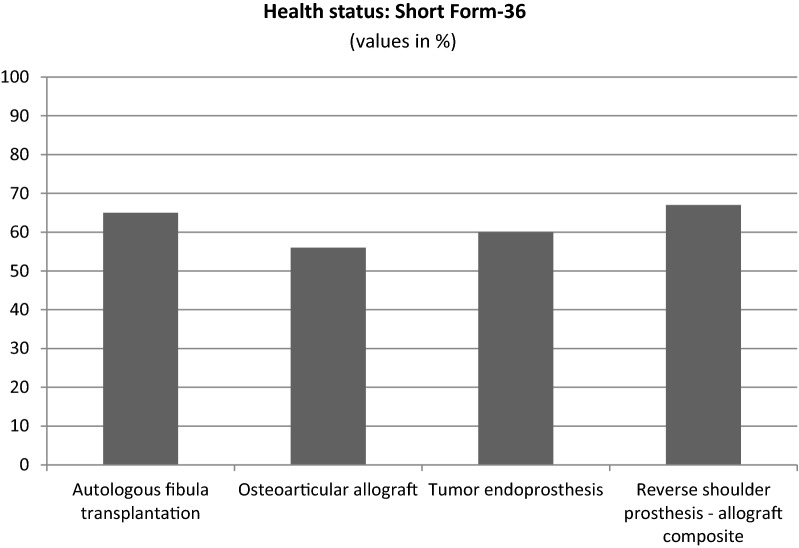
Based on the results from the SF-36 questionnaires no significant difference was observed between the groups

Quality of life (health status) was assessed and calculated based on the SF-36 questionnaire. Scores were similar in all 4 groups: 56–67%, no significant differences were found (Fig. 3).

## Discussion

The proximal humerus is the 3rd most common site for primary bone and soft tissue tumors [3]. Salvage of the limb and retaining function present several challenges [4, 8, 14, 20, 24, 26–29]. An oncologically appropriate and neccessary resection leaves not only a significant bone defect, but may also affect both the presence and function of the active and passive stabilizers (i.e. the rotator cuff, the deltoid muscle and the axillary nerve) to a different degree [3, 14, 16, 17, 20, 23, 31]. Several procedures have been implemented to reconstruct the bone defect and to restore function [5, 6, 8, 11, 14, 20, 21, 30]. The results of different studies from the relevant literature are summarized in Table 3; [1, 4, 16, 19, 20, 29, 31, 32].

**Table 3:** Clinical data, functional scores and complications of different reconstuction techniques collected from the literature

Study	Technique	Number of patients	Mean follow-up (months)	Active abduction-elevation	Mean MSTS score	Complication rate	Complication
Cannon et al [1]	Endoprosthesis	83	30	41°	63%	29%	Proximal migration: 22
De Wilde et al. [3]	Reverse shoulder prosthesis	14	91	157°	Not reported	28%	Dislocation: 2 Infection: 2
El Sherbiny et al. [5]	Free vascularized fibula, lateral scapular crest flap, endoprosthesis	32	19–92	20°–30°	FVFib–72% LSCrFl − 68% Endoprosthesis 71%	18–25%	Nonunion, proximal migration
Griffiths et al. [9]	Endoprosthesis	42	71	Not reported	72, 3% and 77, 7% (with constrained liner)	31%	Infection: 2 Dislocation: 14
Kaa et al. [16]	Reverse shoulder prosthesis	16	46	78°	77%	37%	Infection: 3 Aseptic loosening: 2 Dislocation: 1
Lazerges et al. [18]	Composite reverse shoulder arthroplasty	6	71	57° Glenohum. Abduction	73%	No complication in 6 patients	None
Raiss et al. [11]	Endoprosthesis (mutars)	39	38	33°	63%	18%	Infection: 2 Dislocation: 5
Ruggieri et al. [32]	Allograft–resurfacing composite	14	25	Not reported	77% (46, 7–86, 7%)	21%	Fracture: 3
Sande et al. [19]	Endoprosthesis (modular) allograft–prosthesis comp., osteoartic. alograft	14 10 13	120	Not reported	77, 72 and 76%	21, 60 and 0%	Inf: 1 Fract: 1 Disloc: 1 Inf: 1 Fract: 1 Disloc 3 Inf: 0 Fract: 0 Disloc: 0
Streitbuerger et al. [31]	Reverse shoulder prosthesis (mutars)	18	20	78°–88°	24–25%	29%	Infection: 1 Dislocation: 4
Trovarelli et al. [20]	Reverse shoulder prosthesis (mutars)	28	60	40°–180°	29%	27%	Dislocation: 5 Aseptic loosening: 1
Yang et al. [13]	Endoprosthesis, devitalised bone grafts, osteoartic. allograft, autologous fibula	35	71	Not reported	77%	17%	Infection: 4 Fracture:1 Graft resorption: 1
Authors results	Autologous fibula transplant. Osteochondral homograft transplant. Tumor endoproth. hemiarthroplasty Reverse prosthesis-allograft comp	25 10 43 11	96	50°–80°, 30°–50°, 30°–60° and 120°–160°	70, 64, 67 and 84%	24, 40, 14 and 54%	Fracture: 6 Fracture: 3 Infection: 1 Dislocation: 5 Infection: 1 Dislocation: 6 Infection: 0

In our retrospective study, 90 patients underwent one of four different types of limb-salvage surgery: autologous fibula or osteoarticular allograft transplantation, hemiarthroplasty with a tumour endoprosthesis, and reverse shoulder prosthesis—allograft composite reconstruction. The type of surgery was not randomly assigned, but was chosen based on the following criteria: in younger patients, who had small sized humeri, we aimed to reconstruct the defect with an autograft. In cases where life expectancy was poor (metastasis), the patients were older, or the defect was large, we preferred to perform a hemiarthroplasty using a tumor endoprosthesis, or on occasion reconstruction using a homograft. As reverse shoulder prostheses implantation gained popularity in the past decade, we started to perform conventional hemiarthroplasty with a reverse prosthesis-homograft composite more and more. Intact delta muscle innervation, and saving two-thirds of the deltoid muscle belly are pre-requisites for this procedure [10, 12, 14, 15, 20].

Twenty five (28%) of the 90 patients underwent autologous fibula transplantation, in 2 cases vascularized fibula was used. The incidence of the most common complication—fracture of the graft—was high, and amounted to 24% in our series. Even if a vascularized fibula is used fractures and development of a pseudoarthrosis of these grafts is common, this is described in the literature as well (24–27%) [13, 33]. Fracture healing was usually reliable following conservative treatment, though data from the literature suggests that half of these patients are bound to undergo at one more surgrical session [33]. Range of motion values differed significantly and this was affected largely by whether the fibula fracture healed or there was a pseudoarthrosis. Mobility was excellent in former cases and much worse in the latter, but was overall better compared to the results from the hemiarthroplasty group.

In terms of function, MSTS scores were 70% on average in the fibula transplantation group, corresponding well to the data from the literature suggesting values between 63 and 78% [13]. This biological reconstruction has proven to be reliable and is our preferred method in pediatric cases (children between ages 6–10 years) where the anatomical situation does not permit the implantation of either a tumor or a reverse shoulder endoprosthesis.

While the major concern regarding fibular grafts is fracturing, main concerns for the osteoarticular allografts are septic complications, development of a pseudoarthrosis, partial resorption/collapse of the allograft and implant fracture [17, 33].

In our series 4 of the 10 osteochondral massive proximal humerus allografts failed and further surgical management was neccessary. High complication rates regarding allografts are also reported in the literature [19, 30]. Whereas Abdeen et al. [8] suggest that a prosthesis-allograft composite provides greater mechanical solidity, and a more physiological opportunity for ligament and tendon reconstruction, Streitbürger et al. do not recommend using massive allografts, or prosthesis-allograft composite solutions on the upper limb [31]. DeGroot et al. [30] deem osteochondral allografts to be the best reconstructive option, and attempt to prevent fractures by inserting bone cement into the graft. However, even with these precautions fractures occur in almost 20% of cases [30]. We found that even though we aim to preserve and reconstruct the joint capsule and the tendons of the rotator cuff, we did not observe beneficial effects on joint function. We also found MSTS scores to be the lowest in this group, namely 64%. MSTS scores in the allograft group are reported to be around 70–80% in the literature [14, 18, 20, 32]. The high complication rates, poor range of motion values and consequential poor shoulder joint function that we ourselves encountered lead us to discontinue solitary allograft implantation years ago.

Reconstruction of the proximal humerus with a modular tumor endoprosthesis in the form of hemiarthroplasty has proven to be a reliable method with low-complication rates over the decades [1, 14, 17, 19, 27, 32]. The surgical procedure itself is less demanding and operative time is shorter than regarding the other three interventions. It is the preferred method when treating bone metastases, or even pathological fractures [12, 21, 26]. The disadvantage of performing a hemiarthroplasty by using a tumor endoprosthesis is the poor range of motion in the shoulder even when the rotator cuff may be salvaged; in these cases the prosthesis functions as a spacer. Moreover, stability of the shoulder joint will often be jeopardized by sacrificing the axillar nerve which often leads to subluxation of the prosthesis caudally or due to marked delta muscle activity (migration of the prosthesis proximally) [1, 12, 34]. Attempts should be made to prevent this complication by using different artificial ligaments (we used the trevira attachment tube) (Implantcast Gmbh) [34], but it did not provide protection in all of our cases. Others found [14, 26, 35] that the trevira attachment tube is a valuable tool in the fixation of the soft tissues surrounding the prosthesis and it’s use improves function as well. MSTS scores have been reported to be around 63–75% following tumour prosthesis hemiarthroplasty [1, 9, 11, 17], according to our observations (67%).

The functional deficit caused by the absence of the rotator cuff may be compensated for by implanting a reverse total shoulder prostheses used routinely in rotator arthropathy and deficiency [12, 20, 36, 37]. These prostheses are semi-constrained, and the distal and medial shift in the pivot point of the joint allows for a surprisingly good range of motion and function driven by the deltoid muscle only [12, 20, 31, 38]. Even though short- and midterm results are promising, the data and experience available regarding the implantation of these types of prostheses in tumor cases––especially in combination with allografts—is limited [3, 10, 16, 18, 20]. We were able to follow-up 12 patients who had undergone reverse prosthesis-allograft composite implantation for over five years. The best MSTS scores were recorded in this group, and were similar to 80% MSTS results recorded in the literature regarding reverse prostheses [3, 10, 15, 16, 20]. According to our results this procedure ensures good function and patients compliance according MSTS and SF-36 scores. Although instability was the only complication in the early postoperative period, it could be easily managed conservatively in most cases. According to our experience a 6 week course of immobilization is usually sufficient for the recovery of deltoid muscle and the surrounding soft tissues following closed reposition [10, 15, 16]. As the delta muscle is the main motor of the prosthesis it’s presence is mandatory for reasons of stability if resection of the tumor permits sparing the attachment of the muscle on the deltoid tuberosity. We encountered instability in the cases where resection was performed below this area, or if the axillary nerve was either injured or needed to be sacrificed even partially. The allograft serves as an attachment site for the soft tissues. We found that with the proper indication the reverse shoulder prosthesis-allograft composite implantation is a preferable procedure in bone tumors. The merits of this procedure are verified by long-term follow-up [15, 16].

We assumed a string correlation between shoulder mobility and patient satisfaction evaluated by different scores. However, this was not reflected when evaluating the functional MSTS scores, or the short-Form 36, as overall patient satisfaction was nearly equally good in all of the proximal humerus reconstruction groups. An underlying explanation may be that patient satisfaction regarding the upper limb function evaluated by these scores was not only dependent on shoulder range of motion, but also on other relevant factors, i.e., painless motion, stability of the shoulder joint. Furthermore this reduced range of motion is nearly enough for everyday activities.

Strengths and limitation of the present work: The evaluation period spans over three decades, reconstructive surgerical options have changes significantly leading to changes regarding indications for each procedure. Today in certain cases we would be more likely to implant a reverse shoulder endoprosthesis than a homograft. A strong point of the study on the other hand is that we were able to evaluate a significant number of different procedures which allowed us to compare different complications and to focus on patient satisfaction and quality of life using regarding these four techniques.

## References

[1] Cannon CP, Paraliticci GU, Lin PP (2009). Functional outcome following endoprosthetic reconstruction of the proximal humerus. J Shoulder Elbow Surg.

[2] Teunnis T, Sjoerd PFT, Hornicek FJ (2014). Outcome after reconstruction of the proximal humerus for tumor resection: a systematic review. Clin Orthop Relat Res.

[3] Wilde De, Boileau PF, van derBracht H (2011). Does reverse shoulder arthroplasty for tumors of the proximal humerus reduce impairment?. Clin Orthop Relat Res.

[4] Dieckmann R, Liem DF, Gosheger GF (2013). Evaluation of a reconstruction reverse shoulder for tumour surgery and tribological comparision with an anatomical shoulder arthroplasty. Int Orthop.

[5] El Sherbiny M (2008). Reconstruction of the proximal humerus after wide resection of tumors: comparison of three reconstructive options. J Egypt Natl Cancer Inst.

[6] Gosheger G, Hardes JF, Ahrens HF (2005). Endoprosthetic replacement of the humerus combined with trapezius and latissimus dorsi transfer: a report of three patients. Arch Orthop Trauma Surg.

[7] Wafa H, Reddy KF, Grimer RF (2014). Does total humeral endoprosthetic replacement provide reliable reconstruction with preservation of a useful extremity?. Clin Orthop Relat Res.

[8] Abdeen A, Hoang BH, Athanasian EA (2009). Allograft-prosthesis composite reconstruction of the proximal part of the humerus: functional outcome and survivorship. J Bone Joint Surg Am.

[9] Griffiths D, Gikas PD, Jowett CF (2011). Proximal humeral replacement using a fixed-fulcrum endoprosthesis. J Bone Joint Surg Br.

[10] Grosel TW, Plummer DR, Everhart JS (2019). Reverse total shoulder arthroplasty provides stability and better function than hemiarthroplasty following resection of proximal humerus tumors. J Shoulder Elbow Surg.

[11] Raiss P, Kinkel S, Sauter UF (2010). Replacement of the proximal humerus with MUTARS tumor endoprostheses. Eur J Surg Oncol.

[12] Sirveaux F (2019). Reconstruction techniques after proximal humerus tumour resection. Orthop Traumat Surg Res.

[13] Yang Q, LiYang JFZF (2010). Limb sparing surgery for bone tumours of the shoulder girdle: the oncological and functional results. Int Orthop.

[14] Nelson Fred RT (2019). What is the optimal reconstruction option after the resection of proximal humeral tumors? A systematic review. Open Orthop J.

[15] Houdek MT, Bukowski BR, Athey AG, Elhassan BT (2020). Comparison of reconstructive techniques following intraarticular resection of proximal humerus. J Surg Oncol.

[16] Kaa AKS, Jorgensen PH, Sojbjerg JO (2013). Reverse shoulder replacement after resection of the proximal humerus for bone tumours. Bone Joint J.

[17] KissJ SG, Antal I (2007). Functional results and quality of life after shoulder girdle resections in musculoskeletal tumors. J Shoulder Elbow Surg.

[18] Lazerges C, Dagneaux L, Degeorge B (2017). Composite reverse shoulder arthroplasty can provide good function and quality of life in cases of malignant tumour of the proximal humerus. Int Orthop (SICOT).

[19] Sande MAJ, Dijkstra PDS, Taminiau AHM (2011). Proximal humerus reconstruction after tumour resection: biological versus endoprosthetic reconstruction. Int Orthop.

[20] Giulia T, Capellari A, Angelini A, Elisa P, Ruggieri P (2019). What is the survival and function of modular reverse total shoulder prostheses in patients undergoing tumor resections in whom an innervated deltoid muscle can be preserved?. Clin Orthop Rel Res.

[21] Capellari A, Trovarelli G, Crimi A (2020). New concepts in the surgical treatment of actual and impending pathological fractures in metastatic disease. Injury.

[22] Malawer MM, Meller I, Dunham WK (1991). A new surgical classification for shoulder––girdle resections: analysis of 38 patients. Clin Orthop Relat Res.

[23] Brazier JE, Harper R, Jones NMB (1992). Validating the SF-36 health survey questionnaire: new outcome measure for primary care. BMJ.

[24] Enneking WF, Dunham W, Gebhardt MC (1993). A system for the evaluation of reconstructive procedures after surgical treatment of tumors of the musculosceletal system. Clin Orthop Relat Res.

[25] Henderson ER, O’Connor MI, Ruggieri P, Windhager R, Funovics PT, Gibbons CL, Gou W, Hornicek FJ, Temple HT, Letson GD (2014). Classification of failure of limb salvage after reconstructive surgery for bone tumours. Bone Joint J.

[26] Mourikis A, Mankin HJ, Hornicek FJ (2007). Treatment of proximal humeral chondrosarcoma with resection and allograft. J Shoulder Elbow Surg.

[27] Piccioli A, Maccauro GF, Rossi BF (2010). Surgical treatment of pathologic fractures of humerus. Injury.

[28] Wieser K, Modaressi K, Seeli F (2013). Autologous double-barrel vascularized fibula bone graft for arthrodesis of the shoulder after tumor resection. Arch Orthop Trauma Surg.

[29] Wang Z, Li JF, Guo ZF (2010). Functional outcomes and complications of reconstruction of the proximal humerus after intra-articular tumor resection. Orthop Surg..

[30] DeGroot H (2004). The use of cement in osteoarticular allografts for proximal humeral bone tumors. Clin Orthop Relat Res.

[31] Streitbuerger A, Henrichs M, Gosheger G, Ahrens H, Notrott M, Guder W, Dieckmann R, Hardes J (2015). Improvement of the shoulder function after large segment resection of the proximal humerus with the use of an inverse tumour prosthesis. Int Orthop.

[32] Ruggieri P, Mavrogenis AF, Guerra GF (2011). Preliminary results after reconstruction of bony defects of the proximal humerus with an allograft-resurfacing composite. J Bone Joint Surg.

[33] Zelenski N, Brigman BE, Levin LS (2013). The vascularized fibular graft in the pediatric upper extremity: a durable, biological solution to large oncologic defects. Sarcoma.

[34] Ahrens HF, Hardes J, Nottrott MF (2012). Attachment tube for soft tissue reconstruction after implantation of a mega-endoprosthesis. Oper Orthop Traumatol..

[35] Wang B, Wu Q, Liu J, Yang S, Shao Z (2014). Endoprosthetic reconstruction of the proximal humerus after tumour resection with polypropylene mesh. Int Orthop.

[36] Lädermann A, Edwards TB, Lädermann G (2014). Arm lengthening after reverse shoulder arthroplasty. Int Orthop.

[37] Degeorge B, Chammas M, Coulet B (2020). Allograft-composite reverse shoulder arthroplasty for malignant tumor of the proximal humerus. Tech Hand Upper Extremity Surg.

[38] Scarlat MM (2013). Complications with reverse total shoulder arthroplasty and recent evolutions. Int Orthop.

